# Lectures on the Comparative Anatomy of the Vertebrate Animals

**Published:** 1847-07-01

**Authors:** 


Lectures on the Comparative Anatomy and Physiology of the Vertebrate Animals; Delivered at the Royal College of Surgeons of England in 1844 and 1846. By *Richard Owen*, F.R.S., Hunterian Professor and Conservator of the Museum of the College. Part I.—Fishes. Illustrated by numerous Woodcuts. 8vo. Longman and Co. 1846.

The second volume of this important work has now made its appearance, and calls for an early notice at our hands. It may be considered as by far the most important of the series, as it enunciates the new and profound views of its distinguished author on some of the highest and most abstruse theories of transcendental anatomy. It is for this reason that we now propose to take up this volume for our separate consideration, not associating it, in our notice, with the former one, nor waiting until the work shall be completed by the publication of the third and concluding part.

The promulgation of theories, and many of them highly ingenious and even exhibiting no small degree of philosophical generalization, in the structure of particular groups of animals, is not a new thing. The first, however, who carried his views of the subject into anything like a full and consistent plan, was Geoffroy St. Hilaire; whose genius, as brilliant as it was bold, gave to his theories a character of deep and varied interest, by which the philosophical world was equally astonished and charmed. The fascination which belonged to his splendid and glorious idea (and we feel no hesitation in designating it by so strong a term,) the charm which always attaches to a single, uniform and all-inclusive theory dazzled his first disciples, and prevented them from seeing the inconsistencies and incongruities which in truth were included in it, whilst those to whose clearer vision these faults were apparent, became the founders or the adherents of an opposing party, who rejected the principle on account of its defects, and fell into a greater error in endeavouring to escape from the less.

Passing the clever vagaries of Carus, and the hypotheses of many others of various degrees of demerit, we believe that it was left to Professor Owen, by the enunciation of a clear and tangible theory, by the discrimination between those structural elements which are essential to the relation of beings of the same type, and those which only subserve a definite and, as it were, individual purpose, to exhibit the true conditions of that beautiful and unerring law which provides for the various requirements of every subordinate group or species, by modifications of the typical structure, rather than by the loss of any of its essential elements and the substitution of others foreign to it; whilst it recognises, in certain subordinate structures which have hitherto been confounded with the former essential and typical ones, a certain superadded and non-essential development of abnormal elements. To these two distinct classes the author gives the terms—somewhat pedantic perhaps, but sufficiently expressive—of *homological* and *teleological* structures.

Before we advance, however, to the more detailed development of this theory, we will allow the author to exhibit his general views on the structure of the great division of the animal kingdom, the Vertebrata, on a portion of which the present volume treats.

We are met, *in limine,* by an expression which we confess we do not fully understand; are we to consider it as declaring the deliberate judgment of the author on the comparative merits of the different objects of study and research there mentioned? The passage to which we allude is as follows :—

"And first, permit me to dwell a little on the inestimable privilege which we enjoy, in entering upon our professional studies by the portal of anatomy.

"How vast and diversified a field of knowledge opens out before us as we gaze from that portal! Consider what it is that forms the subject of our essential introductory study; nothing less than the organic mechanism of the last and highest created product which has been introduced into the planet; *contrast this, which both Sage and Poet have called 'the noblest study of mankind,' with the dry and unattractive preliminary exercises of the Lawyer or the Divine.*"

Indeed! is this Professor Owen's deliberate opinion? We are old-fashioned enough to think that there may be a still nobler study to which the mind, the heir of immortality, may be devoted. We have fancied, without any derogation from the value and importance of the casket, that the gem contained within it was still more worthy of contemplation—that the value and interest of the body depend mainly on its being the seat of the immortal part of our nature, and that the divine emanation by which the corporeal form is animated and ennobled, may afford subject for a higher and even more interesting investigation, than the material of which we are composed. But let this pass.

The following statement of the author's views on a theory, the truth of which has for some years been too generally and readily taken for granted, will be read with interest, although we could have wished it to have been more fully developed.

" In ascending to man, we trace a very extensive and varied but progressive course of development, through the great vertebrated series which commences at a very low point.

" It might, perhaps, be imagined that the lowest vertebrate forms began where the highest invertebrated form ended, and made a direct step in advance in the scale of animal organization. Such, indeed, ought necessarily to follow on the hypothesis of the development of species by progressive transmutation, and of the arrangement of animal life in a single and uninterrupted chain of being.

" But truer views of the nature and direction of Zoological affinities, and a deeper insight into the laws of Development and of Unity of Organisation in the Animal Kingdom, concur to disprove the once favourite and recently-revived hypotheses. We have seen that the Invertebrata resemble each other only at the earliest and most transitory periods of their development, diverging thence, in special directions, to the manifestation of very distinct types of animal structure. So likewise we must look to the very beginning of the development of the Vertebrate animal before we shall discover that amount of concordance which will justify us in predicating 'Unity of Organisation' between it and any of the Invertebrated forms. And when, with infinite care and minutest scrutiny, availing ourselves of all the aids and appliances of optical art, we have arrived at clear and satisfactory demonstration of the greatest amount of resemblance, in constitution and properties, between the Vertebrate embryo and the Invertebrate adult,—it is not with any of the higher forms of Invertebrata,—with neither the Cephalopod, the Arachnidan, nor the Insect,—that such organic correspondence is found to exist; but it is with the lowest forms and simplest beginnings of animal life,—with the infusorial monads. Only, in fact, during that period of the ovum-life of the Vertebrated being, in which the mysterious properties of the impregnated germ-vesicle are diffused and distributed by fissiparous multiplication amongst countless nucleated cells—the progeny of the primary germinal vesicle and coheirs of the seminal virtue—do we find such a form and such properties of the Vertebrated animal as justify us in affirming that there is 'Unity of Organisation' between it and an Invertebrate animal." P. 11.

A succinct and well-arranged account of the general structural characters of the vertebrate classes occupies the principal portion of the first lecture, which however does not exhibit any novelty in generalization or detail to warrant us in delaying our advance to the next lecture, in which the author's original views on the homologies of osteology are first enunciated. The following passage, which commences this portion of the work, exhibits the extensive range of organs which the author considers as comprised within the definition of the term skeleton.

" The branch of anatomy which treats of the Skeleton of Vertebrate animals is designated 'Osteology,' because in anthropotomy it relates exclusively to the bones and teeth. But the skeleton, according to its etymological signification of hard and dry parts, might apply to the hair and nails, and, indeed, the entire epidermal system. When, also, in a general survey of the Vertebrata, we see the spinal column gristly in some fishes, and the tendons bony in some birds; and when we call to mind such homological relations as that of the fibro-membranous sclerotic of the human eye with the cartilaginous sclerotic of the turtle and the osseous sclerotic of the cod-fish, it will be obvious that the present branch of anatomy ought naturally to embrace the aponeuroses, ligaments, and cartilages, since these are so many arrested stages in the histological development of the internal skeleton." P. 20.

We confess that we dissent from this extended view of the term, as being absolutely indefinite; and the examples just cited by Professor Owen only appear to us to exhibit the difficulties of a definition without solving them. The distinction, however, which he has drawn, in the former volume of his work, between the terms analogue and homologue, and the subsequent development of his theory of homological and teleological relations, shew that the difficulty is confined to words, and that the author's views are clear, distinct, and definite.

The hypothesis of Geoffroy St. Hilare, of the theoretical identity of the crust or exo-skeleton of the annulosa and the endo-skeleton of the vertebrata, an hypothesis which involved the author in such a labyrinth of contradictions, as required a genius like his to give it even the colour of consistency, has at present but few supporters. The application of the term skeleton, indeed, to such parts as the skin of the annelides, and the polypary of the corals was enough to startle those who had been accustomed to the older restriction of the term to the actual bony structures of the vertebrata, and the endeavour to prove the homologies (to apply a new term to an old idea) between the rings of the articulata, the scutum of the cephalopoda, and the vertebral column of the highest grand division of the animal kingdom, was sufficient to split the scientific world at once into two great parties; those who looked upon this great naturalist as an unerring guide, the Newton of transcendental anatomy, and those who considered him as a mere visionary, ingenious indeed and full of talent, but still altogether a visionary. On this subject Professor Owen has the following pertinent remarks:—

"In the Invertebrata we saw that the skeleton, or parts analogous to the bones of the Vertebrata, commonly consisted of large, strong, thick, often unjointed plates, developed in or upon the skin, hardened principally by carbonate of lime, protecting the whole body, and having the muscles attached to the inner surface.

"In the Vertebrata the skeleton chiefly consists of diversely configurated, but most commonly cylindrical and articulated pieces, hardened chiefly by phosphate of lime, developed from fibrous and cartilaginous tissue in the interior of the body, of which it forms the internal framework, giving attachment to the muscles by the outer surface, and subserving their action as levers and fulcra.

"The exterior calcified shells and crusts of the Invertebrata are unvascular; they grow by the addition of layers to their circumference, or they may be cast off when too small for the growing body, and be reproduced of a more conformable size; but they have no inherent power of repair.

"The internal bones of the Vertebrata are vascular; they grow by internal molecular addition and change, and have the power of repairing fracture or other injury.

"Such are the broad and obvious distinctive characters of the skeletons of the Invertebrate and Vertebrate animals; the contrasts having relation chiefly to the difference in the development of the nervous system. Thus, when the powers of discerning and avoiding lethal or hurtful agencies are dull and contracted, the entire animal is protected by a hard insensible dermal armour, or exo-skeleton; but, as those powers become expanded and quickened, the body is disencumbered of its coat of mail, the skeleton is put inside, and made subservient to the activities, and the skin becomes proportionally more susceptible of outward impressions of pleasure and pain.

"Some estimable anatomists, who have more especially devoted their attention to the detection of the corresponding parts in different animals, have supposed that these different functions were performed by modifications of essentially the same or homologous parts of the skeleton.

"Observing that a segment of the outer skeleton of an articulate animal, the thoracic ring of a lobster for example, formed a small canal for the nervous trunks, and a larger one for the vascular trunks and plastic organs; and that a thoracic segment of the skeleton of a Vertebrate animal also formed a small protecting canal for the spinal chord, and a larger hoop about the vascular and other viscera of that cavity,—they have concluded that both were modifications of the same elements or primary segment of the skeleton. Carus, for instance, calls both rings 'vertebræ;' and Geoffroy St. Hilare thought it needed but to reverse the position of the Crustacean,—to turn what had been wrongly deemed the belly upwards,—in order to demonstrate the unity of organisation between the Articulate and Vertebrate animal. But the position of the brain is thereby reversed, and the alimentary canal still intervenes in the Invertebrate between the aortic trunk and the neural canal.

"The outer and the inner skeletons do agree in certain relations; neither of them are primitive parts of the organism, but are modifications or metamorphoses of other pre-existing systems: both serve as fixed points of attachment to the muscles, aid their action as levers, and determine the kind of movements by particular joints: both are organs of protection and support.

"But, besides the differences of tissue, mode of growth and vital properties, already noticed, the exo- and the endo-skeletons differ in the one being developed from the skin, the other from the internal cellular and fibrous systems. The exo-skeleton defends or surrounds the periphery of the animal; the endo-skeleton the internal parts. The exo-skeleton is related to the muscles by its inner surface, the endo-skeleton by its outer surface. The exo-skeleton is the reflex of the circumambient medium and relations of the animal: the endo-skeleton is the index of its motive energies and its intelligence." P. 22.

And he very properly adds—

"Only the highest of the Mollusca possess a true homologue of the endoskeleton, developed in relation to the defence of the nervous centres: but it is a feeble cartilaginous rudiment in the best organised Cephalopods; and, in the cuttle-fish, is far outweighed by the calcareous dorsal plate which still represents the exo-skeleton of the testaceous mollusks. Thus a cartilaginous cranial vertebra co-exists in the highest Invertebrata with a calcareous dermal skeleton; and there is no abrupt contrast in passing thence to the consideration of the skeleton in the Vertebrata." P. 23.

The existence of an exo-skeleton is recognised, indeed, by our author in the vertebrata in the scales of fishes in all their modifications, in those of serpents and lizards, in the ossified scales of the crocodile, and the horny plates of the tortoise, the "tesselated armour" of the armadillo, the imbricated pointed scales of the manis, the spines of the hedgehog, the quills of the porcupine, the feathers of the bird, and the hair of the ordinary quadruped.

In treating of the structure of true bone, the following passage contains Mr. Owen's opinions on this important point in structural anatomy.

"The blastema or primitive basis of bone is not originally cartilage, but more resembles mucus in its chemical characters: it appears at first to be a subtransparent glairy fluid, but contains a multitude of minute corpuscles. Its assumption of the cartilaginous character and consistency is attended with the appearance in it of numerous small, sub-elliptic, nucleated cells. As the cartilage hardens, these cells increase in number and size, and begin to accumulate, and to be arranged in linear series at the part where ossification is about to commence. These series in the cartilage of long bones are usually vertical to its ends, and in flat bones are vertical to the peripheral edge; *i.e.* they are parallel to the axis of the long bone, and are radiated in the flat one, but not with mathematical exactness.

"The nucleated cells are the instruments by which the earthy particles are arranged in order; and, in bone, as in tooth, there may be discerned in this predetermined arrangement, the same relation to the acquisition of strength and power of resistance, with the greatest economy of the building material, as in the disposition of the beams and columns of a work of human architecture.

"The power of the cells so to operate upon the salts of the plasma, which percolates the intervening tissue, seems to reside chiefly in the repellent property of their nuclei: I have been led by observation of some of the phenomena of osteogeny to surmise that the walls and the nucleus of the cell were in opposite electric or magnetic states, one attracting, the other repelling, the surrounding earthy particles.

"Certain of the columnar series of nucleated cells become more aggregated or pressed together; their nuclei become more concentrated, and, according to Miescher and Gerber, they coalesce and become dissolved, leaving a cylindrical tube, parallel with the long axis of the future bone. First, a reddish lymph, and then a capillary vessel is prolonged into each of these cylinders, which is converted into a 'Haversian,' or vascular canal: before, however, the direct influence of the circulation has penetrated so far, the nucleated cartilaginous cells have arranged or propagated themselves in concentric series round the cylinder, and the intervening layers of the molecular blastema begin to be impregnated with the hardening salts, which, being repelled by the nuclei of the cells, are forced into the concentric laminated arrangement around the Haversian canal. The establishment of the capillary circulation in these canals accelerates the progress of ossification by the rapid import of new material: the resisting nuclei of the surrounding concentric cells, pressed on all sides, undergo a remarkable change, and the nucleolar matter is forced out in rays, but chiefly in the direction where the resistance is least, viz. towards the Haversian canal. The remaining central nuclear matter and that of the diverging rays finally become dissolved, and establish permanent bone-cells and minute tubes, which tubes, traversing the concentric lamellæ, open into the Haversian canal, and receive the transuded plasma from the blood-capillary. The tubes branch and anastomose, and form the medium of the transmission of the plasma through the densest osseous tissue." "I have detected a similar system of plasmatic tubes in tendon: and they probably exist under characteristic modifications in all tissues constituting the essential nutritive system of each."

The true nature of the "metamorphosed cartilaginous cells in bone" is maintained by Professor Owen to be cells or lacunæ filled with earthy salts; in which opinion he follows Müller. It is indeed surprising how any one, who has carefully examined these little "radiated corpuscles," could have come to the conclusion that they were either empty lacunæ, as some have maintained, and still do maintain, or cavities filled with fluid, or, in fact, anything but what Müller and Owen and others have declared them to be. The term *corpuscula ossea*, applied to them by Purkinje, is indeed as incorrect as that of empty lacunæ. The solid white reflection from them, when viewed by reflected light, and the dense opacity which is presented by transmitted light, are surely appearances only to be accounted for by this view of their nature; and it is amply borne out by the fact first observed by Professor Müller, that by the action of dilute acid the dark opacity is removed when observed under the latter circumstances, and the bright whiteness under the former. But we must pass over a very interesting detail of some of the principal modifications in the structure of the bones in different animals, to the consideration of the important question propounded by our author in the words "what is a bone?"

This question will not perhaps strike an ordinary human anatomist as one of any great difficulty. He will probably be quite satisfied with the simple definition that "*a bone is any single piece of osseous matter entering into the composition of an adult skeleton;*" which it is obvious is very little more definite than to say that a bone is a bone. Others, perhaps, will consider it sufficient, with Cuvier, to descend to "the primitive osseous centres as they exist in the fœtus."

The former of these definitions requires no refutation; and we refer our readers to Mr. Owen's treatment of the question at p. 36. The definition of Cuvier, however, must not be so passed over. There appears upon the face of it so much plausibility, the idea is in itself so ingenious, and its application so simple, that we are not surprised at the general favour with which it was received, and the hold it has had on the opinions of the most distinguished anatomists. But the consequences of an admission of this theory are such as to carry its refutation with them.

"According to this rule," says Professor Owen, "we ought to count the humerus as three bones, and the femur as four bones instead of one; for the ossification of the latter begins at four distinct points, one for the shaft, one for the head, one for the great trochanter, and one for the distal condyles. But who will be induced to regard these parts and processes as distinct bones? No such distinction is kept up in any of the lower classes. In both Birds and Reptiles the femur is developed from a single centre." P. 38.

The consideration of this question leads the author now to the development of those views to which we have already adverted, and which form, as we confidently believe, the true key to the difficulty. The following passage, for the length of which we do not offer any apology, contains the first distinct enunciation of these views; we allude, of course, to the important distinction between homological and teleological structures in the skeleton.

"The rule laid down by the great French anatomist fails in its application to the difficult point under consideration, because he did not distinguish between those centres of ossification that have homological relations, and those that have only teleological ones: *i. e.* between the separate points of ossification of a human bone which typify permanently distinct bones in the lower animals, and the separate points which, without such signification, facilitate ossification, and have for their final cause the well-being of the growing animal. The young lamb or foal, for example, can stand on its four legs as soon as it is born; it lifts its body well above the ground, and quickly begins to run and bound. The shock to the limbs themselves is broken and diminished at this tender age, by the divisions of the supporting long bones,—by the interposition of the cushions of cartilage between the diaphyses and the epiphyses. And the jar that might affect the pulpy and largely developed brain of the immature animal is further diffused and intercepted by the epiphysial articular extremities of the bodies of the vertebræ.

"We thus readily discern a final purpose in the distinct centres of ossification of the vertebral bodies, long bones, and the limbs of mammals, which would not apply to the condition of the crawling reptiles. The diminutive brain in these low and slow cold-blooded animals does not demand such protection against concussion; neither does the mode of locomotion in the quadruped reptiles render such concussion likely; their limbs sprawl outwards, and push along the body, which commonly trails upon the ground; therefore we find no epiphyses with interposed cartilage at the ends of a distinct shaft in the long bones of Saurians and Tortoises. But when the reptile moves by leaps, then the principle of ossifying the long bone by distinct centres again prevails, and the extremities of the humeri and femora long remain epiphyses in the frog."

"This distinction in the nature and relations of such centres, which is indispensable in the right application of the facts of osteogeny to the determination of the number of essentially distinct bones in any given skeleton, has never been considered, so far as I know, in that application. Some homologists have gone beyond Cuvier, and still more beyond nature, in arguing the number of individual bones, as indicated by the number of separate centres of ossification in the embryo, to be the same in all vertebrate animals; and that they afterwards differed, or seemed to differ, only by reason of the greater or less rapidity or extent of the confluence of those ossific centres or essentially distinct bones.

"This primitive conformity of separate osseous pieces in the vertebrate series holds good, however, only in regard to the separate centres of ossification of those bones of higher animals which have homological relations to the permanently distinct bones of lower species; it by no means applies to those which have merely teleological relations to the species in which they exist." P. 39.

"The great aim of the philosophical osteologist is to determine, by natural characters, the natural groups of bones of which a vertebrate skeleton typically consists; and, next, the relations of individual simple bones to each other in those primary groups, and to define the general, serial, and special homologies of each bone throughout the vertebrate series.

"By general homology I mean the relation in which a bone stands to the primary segment of the skeleton of which it is a part; thus, when the basi-occipital bone (basilar process of the os occipitis in anthropotomy) is said to be the centrum or body of the occipital or posterior cranial vertebra, its *general* homology is enunciated. When it is said to repeat in its vertebra, or to answer to the basi-sphenoid in the parietal vertebra, or to the body or centrum in the atlas, dentata, or any other of the vertebral segments of the skeleton, its *serial* homology is indicated: when the essential correspondence of the basilar process of the occipital bone in Man with the distinct bone called 'basi-occipital' in a Crocodile or Fish is shown, its *special* homology is determined." P. 41.

We have preferred, by these extracts, giving these important views in the words of the author to attempting an abstract of them in words of our own.

The ground being, as it were, thus cleared before him, the author proceeds to propound his theory of the endo-skeleton of the vertebrata, and especially the theoretical structure of a vertebra. It will be readily imagined that it is in the lowest class of the great group in question that the teleological variations of structure are fewest and least considerable, and the homological most obviously and distinctly and consistently developed. It is, in fact, in the fishes that the illustrations of the theoretical vertebrate structure are most striking and simple. A knowledge of the further development of these elements in the higher and more perfect forms is, however, essential to the full comprehension of the theory, and although the permanently separate condition of most of the distinct centres of ossification in this class will afford an important clue to the understanding of the true character of these homologues in the higher classes, yet it will be impossible fully to understand the relations of the various elements to each other without following them upwards to their most complicated and centralised condition in the mammals. It would not be easy to convey the real objects of the author in his description of a theoretical vertebra without reference to the wood-cuts, or some such pictorial illustrations. We will, however, endeavour to afford some idea of the nomenclature employed by him, and of the normal structure of a vertebra according to Professor Owen's idea, by a comparative statement of the names now given to the parts of which a vertebra is essentially composed with those in ordinary use.

Mr. Owen's definition of a vertebra is, "*One of those segments of the endo-skeleton, which constitute the axis of the body, and the protecting canals of the nervous and vascular trunks;* such a segment may also support *diverging appendages.*"

The essential parts of which a vertebra is theoretically composed are, according to Professor Owen, as follows:—" A body or centrum—two neurapophyses—two parapophyses—two pleurapophyses—two hæmapophyses—a neural spine—a hæmal spine."

" These, being usually developed from distinct and independent centres, I have termed 'autogenous' elements. Other parts, more properly called processes, which shoot out as continuations from some of the preceding elements, are termed 'exogenous:' the diapophyses, or upper 'transverse processes,' and the zygapophyses, or the 'oblique' or 'articular processes' of human anatomy.

" The autogenous processes generally circumscribe holes about the centrum, which, in the chain of vertebræ, form canals. The most constant and extensive canal is that formed above the centrum, for the lodgment of the trunk of the nervous system (neural axis) by the parts thence termed 'neurapophyses.' The second canal, below the centrum, is in its entire extent more irregular and interrupted; it lodges the central organ and large trunks of the vascular system (hæmal axis), and is usually formed by the laminæ, thence termed 'hæmapophyses.' At the sides of the centrum, most commonly in the cervical region, a canal is circumscribed by the pleurapophysis or costal process, and by the diapophysis or upper transverse process, which canal includes a vessel, and often also a nerve.

" Thus a typical or perfect vertebra, with all its elements, presents four canals or perforations about a common centre; such a vertebra we find in the thorax of man, and most of the higher classes of Vertebrata, also in the neck of many birds." P. 43.

We cannot enter into the details of all the modifications of these elements. They are elaborately given in the work before us. It is equally impossible to give the particulars of the variations in form which the vertebræ undergo in the class of Fishes; to which part of his subject the author has devoted a large space. It is extremely probable that the general views here adverted to are correct; and that the details themselves will admit of but little objection or cavilling, as far as they refer to the more normal forms of vertebræ. But whether, in the present state of our knowledge, it is possible to follow our author into all the homologies which he believes himself to have demonstrated in the cephalic vertebræ, is another question, and one on which we are scarcely at present prepared to decide. The following sentence, indeed, at once shews the latitude which

Mr. Owen allows himself, in what may be termed the *morphology* of these parts. He is introducing the description of what he believes to be the "neural arches of the cranial vertebræ."

"The first series of endo-skeletal bones constitutes the axis or back-bone of the skull, as the rest of the vertebral neural arches do that of the trunk; and it includes and protects the encephalon or anterior expanded extremity of the great nervous axis. The under and upper parts of the annular segments are commonly formed by single and symmetrical bones, as in the vertebral axis of the trunk: but sometimes, even in the present low class, the expansion of the cranial cavity is accompanied, not only by a transverse development, but also by a median division of the upper piece or key-bone of one or more of the protecting arches." P. 89.

But now we have something like a law developed in the relation between these presumed "neural arches" and the primary divisions of the encephalon.

"Though subject to various degrees of anchylosis, the cranial vertebræ always accord in number with the primary ganglions or divisions of the encephalon in Fishes. For the better understanding of this important relation, I may premise that the brain of Fishes consists of four primary divisions succeeding each other in a linear series horizontally, which, viewed from behind forwards, are :—

"1. The medulla oblongata, with the superimposed cerebellum, or the 'epencephalon.'

"2. The third ventricle, with its upper (pineal) and lower (hypophysial) prolongations, and the superimposed optic lobes, or the 'mesencephalon.'

"3. The cerebrum proper, or 'prosencephalon.'

"4. The olfactory ganglionic or chord-like prolongation of the cerebrum, or 'rhinencephalon.'" P. 89.

Now it must be confessed that, if this relation be consistently borne out by fact, it would of itself constitute a strong *à priori* argument in favour of the general theory.

In the exposition of these cranial vertebræ—which are of course confined to four, in consonance with the cerebral ganglia which they severally protect, and to which they stand in relation—Mr. Owen selects the Cod (*Gadus morrhua*) as an example affording the most complete development of the different elements. It is extremely difficult even to attempt any analysis of the long and elaborate details of this portion of the work ; but we will endeavour to select the mere heads of each, which is as much as our space will admit of. Taking, therefore, the four "primary ganglia" of the encephalon as our argument or text, we find the first of these, the "epencephalon," is encompassed by what is termed the epencephalic arch of the occipital vertebra. The lowest of these, which answers to the *centrum* of the vertebræ, is the *basi-occipital,* which articulates with the atlas. The *ex-occipitals* form the *neurapophyses* of the vertebræ, and answer to the "occipital latéral" of Cuvier and Agassiz. The *neural spine* of the vertebræ is represented by the *supra-occipital,* answering to the "interpariétal" of Cuvier, &c., and the arch is completed by the representatives of the *par-apophyses,* the *par-occipitals* of Owen, the "occipital externe" of Cuvier.

"The second ring of bones, or that which encircles the mesencephalon," belongs to the parietal vertebræ—the *centrum* of which is the *basi-sphenoid* (sphénoide postérieur, "S. principal" C. and A.) The *neurapophyses* of this vertebra consist of the *ali-sphenoids;* ("Grande aile du sphénoïde," C.) The *spine* is considered to be homologically represented by the *parietals,*—ordinarily two in number,—and the *mastoid* bones are the *parapophyses* of this arch.

The frontal vertebra, the neural arch of which is the procencephalic, has for the representative of its *centrum,* the *pre-sphenoid* ("sphénoide principal," C.) Its *neurapophyses* are the *orbito-sphenoids* ("Aile orbitaire," C.) The *frontal* bone ("Frontal principal," C.) forms the *spine* of the vertebra, and the *post frontals* ("Frontal posterieur," C.) complete the ring as the representatives of the *parapophyses.*

The ring of bones which completes the axis of the cranium anteriorly and forms the nasal vertebra, consists of the *vomer* as the *centrum* of the vertebra; the *nasal* bone as its *spine,* and the *prefrontals* ("Frontal anterieur" C.) as the *neurapophyses.* This presumed vertebra is wanting, as it would appear, in parapophyses.

It will thus be seen that there are four vertebræ standing in relation to the four great cephalic ganglia or divisions. The neural arch of the occipital vertebra is called the ex-encephalic arch, and protects the ex-encephalon; the neural arch of the parietal vertebra is termed mesencephalic from its relation to the mesencephalon;—that of the frontal vertebra is the prosencephalic, from its protecting the prosencephalon; and, finally, the arch of the nasal vertebra is in relation to the rhinencephalon or olfactory ganglion.

Omitting the consideration of the "sense capsules," we pass on to the completion of the cranial or cephalic vertebræ, in the inferior or Hæmal arches, as explained by Professor Owen. "These," says our author, "though apparently more numerous than the vertebral centres, correspond with them and the neural arches, and are essentially four in number, in the osseous fishes; viz. the 'palato-maxillary,' the 'tympano-mandibular,' the 'hyoidean,' and the 'scapular.' Most fishes have likewise appendages, which diverge or radiate from these arches. A special (visceral) system of bony arches, called 'branchial,' also persists in fishes, for the support and movement of the gills." P. 104.

In describing the details of the hæmal arches of the vertebræ, of which we have already attempted a brief analysis of the neural or superior arches, we find, in the present work, the order reversed; and we have to commence in the anterior part of the cranium, with the hæmal arch of the "nasal" vertebra. This is the *palato-maxillary* arch, and its formation is thus described:—

"I am induced to regard this as essentially one arch, from its condition in the Lepidosiren and Plagiostomous fishes, and from the circumstance of its being completed or closed at one point only, viz. where the premaxillaries meet or coalesce. The palatine bones are the piers of this inverted arch, and their points of suspension are their attachments to the prefrontals, the vomerine and the nasal bones. The arch is completed by the maxillary and premaxillary bones, the symphyses of the latter forming its apex; and it is inclined forwards, nearly or quite parallel with the base of the skull; which, in most fishes, extends to the apex of the arch, and in some far beyond it, being usually more or less closely attached to it. In air-breathing vertebrates the arch is more dependent, circumscribing below the nasal or respiratory canal. The pterygoid bones project backwards and outwards as the appendages of the palato-maxillary arch. Both maxillary and inter-maxillary bones tend by their peculiar development and independent movement in bony fishes to project freely outwards, downwards, and backwards. We find, at least, that the general form, position, and attachments of the single and simple palato-maxillary arch, in the *Lepidosiren* or *Cestracion*, are represented in most osseous fishes, by their several detached bones, the names of which have been just mentioned." P. 105.

"The *diverging appendage* of the palato-maxillary arch consists, in fishes, of the pterygoid and ento-pterygoid bones; which, as they are the least important parts of the arch, so are they the least constant." The following sentence shews well the ingenuity and acumen with which Mr. Owen solves difficulties which few men would be disposed to grapple wish.

"The ten bones of which the palato-maxillary arch is composed in Osseous Fishes are, in the Cod and most other species, so dispersed, in relation to the peculiar movements of the mouth, as to appear like three parallel and independent arches, successively attached behind one another, by their keystones, to the fore part of the axis of the skull, and with their piers or crura suspended freely downwards and outwards, except those of the last or pterygo-palatine arch, which abut against the tympanic pedicles. The simplification or confluence of the two first of these spurious arches is effected in the Salmonoid Fishes, by the shortening of the premaxillary, and by the mode of its attachment to the maxillary, which now forms the larger part of the border of the mouth and supports teeth: the maxillaries are brought into close articulation with the palatines in the Plectognathes, and the consolidation of the whole series into its normal unity is effected in the Lepidosiren. The palatines form the true bases of the inverted arch at their points of attachment to the prefrontals; the intermaxillaries constitute the true apex, at their mutual junction or symphysis; the approximation of which to the anterior end of the axis of the skull is rendered possible in fishes, by the absence of any air-passage or nasal canal; the pterygoids are the diverging appendages of the arch; but are attached posteriorly to strengthen the pedicle supporting the lower jaw, and combine its movements with those of the upper jaw; just as the bony appendages of one costal arch in Birds associate its movements with those of the next." P. 110.

The *tympano-mandibular* arch forming the hæmal arch of the frontal vertebra, consists of the *epi-tympanic*, the *meso-tympanic*, the *pre-tympanic*, the *hypo-tympanic*, and the *mandible* or lower jaw; and the *diverging appendage* of this arch "consists of the bones which support the gill-cover, a kind of broad and short fin, the movements of which regulate the passage of the currents through the branchial cavity." These bones are known as the *pre-opercular*, the *opercular*, the *sub-opercular*, and the *inter-opercular*.

The *Hyoidean* arch is regarded as the costal or hæmal arch of the *parietal* vertebra. It is composed of the *stylo-hyal* as the pleurapophysis, the *epi-hyal* and *cerato-hyal* as the hæmapophysis, and the keystone or body of the arch is formed by the *basi-hyals* together with the *glosso-hyal* and *uro-hyal* where these exist. The *diverging appendages* to this arch are the branchiostegous rays attached to the *epi* and *cerato-hyals*.

Those remarkable bony appendages the branchial arches succeed the hyoidean arch, with the key-stone of which they are connected.

The remaining inverted cranial arch, the *scapular*, forms the *hæmal* arch of the occipital vertebra. It is formed by the *suprascapula*, the *scapula*, and the *coracoid;* and its diverging appendage is thus described:

"This appendage consists in Lepidosiren of a single ray; but in Osseous Fishes it is composed usually, first, of two, rarely of three, bones immediately articulated with the coracoid; next, of a series of from two to six smaller bones; which, lastly, support a series of spines or jointed rays. These rays in the scapular appendage, or 'pectoral fin,' are a repetition of the branchiostegal rays in the hyoidean appendage, and of the opercular rays in the tympanic appendage Of the special homology of the pectoral fin-rays with the digits of the pectoral extremity in higher animals, there has been no question. The vegetative repetition of digits and joints, and the vegetative sameness of form in those multiplied peripheral parts of the fins of fishes, accord with the characters of all other organs on their first introduction into the animal series. The single row of fewer ossicles supporting the rays, obviously represents the double carpal series in Mammals; and the bones of the brachium and anti-brachium seem in like manner to be reduced to a single series, unless the humeral segment be confluent with the arch. In the ventral fin no segment is developed between the arch and the digital rays: it is in this respect like the branchiostegal fin." P. 120.

We regret that our limits do not permit our giving an analysis of the author's elaborate exposition of the homologies of the other members in fishes; but we cannot omit his observations on the relations and homologies of the pectoral fin, because they are not only highly interesting in themselves, but they further contribute to the development of those general views and theories which we are now endeavouring to introduce to our readers.

"The special homology of the pectoral fins of fishes with the fore limbs of quadrupeds was indicated by Aristotle, and first definitely pointed out in later times by Artedi, in 1735, who says,—'Ossa pectoris et ventris in piscibus reperiuntur; suntque in piscibus spinosis: 1. Claviculæ; 2. Sternum: 3. Scapulæ, seu ossa quibus pinnæ pectorales ad radicem affiguntur.' (*Partes Piscium*, p. 39). Geoffroy St. Hilaire, who has devoted special Memoirs to the determination of the bones of the pectoral fin, had no knowledge of the primary homology of the pectoral fin as the radiated appendage of the inferior arch of a cranial vertebra, or of its serial homology with the branchiostegal and opercular fins. He consequently speaks of the junction of the basis of the fin to the cranium as something very strange:—"Disposition véritablement très singulière, et que le manque absolu de cou, et une combinaison des pièces du sternum avec celles de la tête pouvoient seuls rendre possible.' (*Annales du Muséum*, IX. p. 361.)

"Oken's latest idea of the essential nature of the arms and legs is, that they are no other than 'liberated ribs.' 'Freye Bewegungsorgane können nichts anderes als frey gewordene Rippen seyn.' (*Lelhrbuch der Natur Phiosophie*, p. 330. 8vo. 1843.)

"Carus (I.), in his ingenious endeavours to gain a view of the primary homologies of the locomotive members, sees in their several joints repetitions of vertebral bodies (*tertiar-wirbel*)—vertebræ of the third degree—a result of an ultimate analysis of a skeleton pushed to the extent of the term 'vertebra' being made to signify little more than what an ordinary anatomist would call a 'bone.'

"But these transcendental analyses sublimate all differences, and definite knowledge of a part escapes through the unwarrantable extension of the meaning of terms. We have seen, however, that a vertebra is a natural group of bones, that it may be recognised as a primary division or segment of the endoskeleton, and that the parts of that group are definable and recognisable under all their teleological modifications, their essential relations and characters appearing through every adaptive mask.

"According to the definition of which a vertebra has seemed to me to be susceptible, we recognise the centrum, the upper (neural) arch, the lower (hæmal)

arch, and the appendages, diverging or radiating from the hæmal arch. The centrum, though the basis, is not less a part of the vertebra, than are the neurapophyses, hæmapophyses, pleurapophyses, &c.; and each of these parts is a different part from the other: to call all these parts 'vertebræ' is in effect to deny their differential and subordinate characters, and to voluntarily abdicate the power of appreciating and expressing them. The terms 'secondary' or 'tertiary vertebræ' cannot, therefore, be correctly applied to the appendages of that natural segment of the endo-skeleton to which the term 'vertebra' ought to be restricted.

"So likewise the term 'rib' may be given to each moiety of the hæmal arch of a vertebra; although I would restrict it to that part of such arch to which the term 'vertebral rib' is applied in Comparative Anatomy and the term 'pars ossea costæ' in Anthropotomy: but, admitting the wider application of the term 'rib' to the whole hæmal arch, yet the bony diverging and backward projecting appendage of such rib or arch is something different from the part supporting it. Arms and legs may be developments of costal appendages, but cannot be ribs themselves liberated: although liberated ribs may perform analogous functions, as in the Serpents and Dragons.

"A series of developments may be traced from the primitive form of the appendage, as a simple plate, spine, or ray, through the many-jointed single ray in the Lepidosiren and the bifurcate jointed ray in the *Amphiuma didactylum*, up to the wing of the Bird and the arm of the Man, without the essential nature of the part being lost sight of; of all these forms of the pectoral member are, in their ultimate or general homology, 'diverging' or 'radiated appendages' of a hæmal arch; but not 'ribs,' nor 'vertebræ.' We may further define the fore-limb, wing, or pectoral fin to be the radiated appendage of the arch called 'scapular,' and this to be the 'hæmal arch of the occipital vertebra.'" P. 126.

In this exposition of the vertebral theory of Professor Owen, it will be seen that considerable support is derived from the simple homologues exhibited in the structure of the *Lepidosiren*, which is here taken for granted to belong to the class of *fishes*. It has always, we confess, appeared to us doubtful whether the reasons elsewhere assigned by Professor Owen for this allocation of the interesting animal in question, be the true one. We have not now space to enter into an elaborate consideration of this point; but we believe we may with less reluctance dispense with the discussion, as it appears from the recent investigations of Mr. John Quekett, on the intimate structure of bone in different classes of the vertebrata, that the *Lepidosiren* is actually a *reptile*, and not a fish. This view is also borne out by many facts in its natural history, and, as we believe, also by its anatomical structure. As far, therefore, as the support derived from this source to the present theories would go, we fear it must be wholly given up.

We have now endeavoured to exhibit, as succinctly as possible, an outline of Professor Owen's views on the homologues of the vertebræ, as exemplified in the osseous fishes, in which the cranial elements are developed in so distinct a manner as to afford the best means of exhibiting their theoretical tendencies. The modifications of these elements as they exist in the so-called cartilaginous fishes, and particularly in the Sturgeon, are extremely interesting, as shewing them in one continuous cartilaginous structure; but it is not necessary here to follow out the elaborate statement of the homological relation of the different portions of this mass of cartilage with the separate elements which we have just enumerated as existing in the Cod. The dermal osteology of the Sturgeon is adduced as illustrative of the author's views, and the median dermal bony plates are considered as "obviously homologous with the dermal bones forming the helmet of the Armadillo, and as bearing the same relation in the Sturgeon to the cartilaginous skull as those bones do in the Armadillo to the osseous skull beneath."

It is not our intention to enter at present into the consideration of the other portions of this elaborate and really valuable work, as our principal object has been to present to our readers some account of the author's theories respecting the homologues of the vertebræ. From even this sketch, we believe it will be seen that it is in the logical exercise of the reasoning faculty that Professor Owen's great strength lies; and although here and there we cannot help fearing that his disposition to theorize may carry him into the mazes of hypothesis, yet, there is so much of sound induction, such clearness in the mode of putting his argument, and so much of the dignity of truth in his conclusions, that no one can rise from the perusal of his works without the conviction that he has been communing with a master-spirit.

The foregoing observations are more particularly borne out in the present work, by that portion of the sixth Lecture which relates to the "Teleology of the Skeleton of Fishes;" and we regret that we cannot present this admirable example of inductive reasoning and eloquent discourse in Natural Theology—for such it may be truly termed—in its completeness. We have no hesitation in saying that the whole of this Chapter, or at least that part of it to which we are now referring, exhibits such a combination of acute reasoning, of close observation, and of appropriate and eloquent diction, as is rarely to be met with within the range of scientific literature. The value of every fact is so carefully weighed, its true bearing upon the question so accurately defined, and the author's theory brought so irresistibly to bear upon the difficulties which former writers have in vain attempted to solve, that scarcely any thing seems to be wanting to render it a perfect example of scientific discussion. We offer a few short passages from it, and with these we conclude our present notice of a work which must be acknowledged fully to confirm the justice of the author's high and extended fame.

"To determine the parts of the Vertebrate skeleton which are most constant; to trace their general, serial, and special homologies, under all the various modifications by which they are adapted to the several modes and spheres and grades of existence of the different species, should be the great aim of osteological science; as being that which will reduce its facts to the most natural order, and their exposition to the simplest expressions. It is impossible, in pursuing the requisite comparisons upwards through the higher organised classes, not to recognise the close and interesting analogies between the mature states and forms of ichthyic organs, and the embryonic condition of the same parts, in the higher species. But these analogies have been frequently overstated, or presented under unqualified metaphorical expressions, calculated to mislead the student and to obstruct the attainment of true conceptions of their nature. We should lose some most valuable fruits of anatomical study were we to limit the application of its facts to the elucidation of the unity of the Vertebrate type of organisation, or if we were to rest satisfied with the detection of the analogies between the embryos of higher and the adults of lower species in the scale of being. We must go further, and in a different direction, to gain a view of the beautiful and fruitful physiological principle of the relation of each adaptation to its appropriate function, and if we would avoid the danger of mistaking analogy for homology or identity, and of attributing to inadequate hypothetical secondary causes the manifestations of Design, of supreme Wisdom and Beneficence, which the various forms of the Animal Creation offer to our contemplation." P. 146.

"Yet there are some who would shut out by easily comprehended but quite gratuitous systems of progressive transmutation and self-creative forces, the soul-expanding appreciations of the final purposes of the fecund varieties of the animal structures by which we are drawn nearer to the great First Cause. They see nothing more in this modification of the skeleton, which is so beautifully adapted to the exigencies of the highest organised of fishes, than a foreshowing of the cartilaginous condition of the reptilian embryo in an enormous tadpole, arrested at an incomplete stage of typical development. But they have been deceived by the common name given to the Plagiostomous fishes: the animal basis of the shark's skeleton is not cartilage; it is not that consolidated jelly which forms the basis of the bones of higher Vertebrates: it has more resemblance to mucus; it requires 1000 times its weight of boiling water for its solution, and is neither precipitated by infusion of galls, nor yields any gelatine upon evaporation.

"In like manner the modifications of the dermal skeleton of fishes have been viewed too exclusively in a retrospective relation with the prevalent character of the skeleton of the Invertebrate animals. Doubtless it is in the lowest class of Vertebrata that the examples of great and exclusive development of the exoskeleton are most numerous; but some anatomists, in their zeal to trace the serial progression of animal forms, seem to have lost sight of all the vertebrate instances of the bony dermal skeleton except those presented by the Ganoid and Placoid fishes. He must have sunk to the low conception that nature had been limited to a certain allowance of the salts of lime in the formation of each animal's skeleton, who could affirm that in the higher Vertebrata 'the internal articulated skeleton takes all the earthy matter for its consolidation,' forgetting that the bulky Glyptodon and its diminutive congeners the Armadillos have their internal skeleton as fully developed and as completely ossified as in any other mammals. The organising energies which perfect and strengthen the osseous internal skeleton do not destroy nor in any degree diminish the tendency to calcareous depositions on the surface, when the habits and sphere of life of the warm-blooded quadruped require a strong defensive covering from that source." P. 148.

"All writers on Animal Mechanics have shown how admirably the whole form of the fish is adapted to the element in which it lives and moves: the viscera are packed in a small compass, in a cavity brought forwards close to the head; and whilst the consequent abrogation of the neck gives the advantage of a more fixed and resisting connection of the head to the trunk, a greater proportion of the trunk behind is left free for the development and allocation of the muscular masses which are to move the tail. In the caudal, which is usually the longest, portion of the trunk, transverse processes cease to be developed, whilst dermal and intercalary spines shoot out from the middle line above and below, and give the vertically extended, compressed form, most efficient for the lateral strokes, by the rapid alternation of which the fish is propelled forwards in the diagonal, between the direction of those forces. The advantage of the bi-concave form of vertebra with intervening elastic capsules of gelatinous fluid, in effecting a combination of the resilient with the muscular power, is still more obvious in the Bony Fishes than in the Shark.

"You may be reminded that all the vertebræ of the trunk are distinct from one another at one stage of the quadruped's development, as in the fish throughout life; and you might suppose that the absence of that development and confluence of certain vertebræ near the tail, to form a sacrum, was a mark of inferiority in fishes. But note what a hindrance such a fettering of the movements of the caudal vertebræ would be to creatures which progress by alternate vigorous inflections of a muscular tail. A sacrum is a consolidation of a greater or less proportion of the vertebral axis of the body, for the transference of more or less of the weight of the body upon limbs organised for its support on dry land; such a modification would have been useless to the fish, and not only useless, but a hindrance and a defect.

"The pectoral fins, those curtailed prototypes of the fore-limbs of other Vertebrata, with the last segment, or hand, alone projecting freely from the trunk, and swathed in a common undivided tegumentary sheath, present a condition analogous to that of the embryo buds of the homologous members in the higher Vertebrata. But what would have been the effect if both arm and fore-arm had also extended freely from the side of the fish, and dangled as a long flexible many-jointed appendage in the water? This higher development, as it is termed, in relation to the prehensile limb of the denizen of dry land, would have been an imperfection in the structure of the creature which is to cleave the liquid element: in it, therefore, the fore-limb is reduced to the smallest proportions consistent with its required functions: the brachial and antibrachial segments are abrogated, or hidden in the trunk: the hand alone projects and can be applied, when the fish darts forwards, prone and flat, by flexion of the wrist, to the side of the trunk; or it may be extended at right angles, with its flat surfaces turned forwards and backwards, so as to check and arrest more or less suddenly, according to its degree of extension, the progress of the fish; its breadth may also be diminished or increased by approximating or divaricating the rays. In the act of flexion, the fin slightly rotates and gives an oblique stroke to the water. For these functions, however, the hand requires as much extra development in breadth, as reduction in length and thickness; and mark how this is given to the so-called embryo or rudimental fore-limb: it is gained by the addition of ten, twenty, or it may be even a hundred digital rays, beyond the number to which the fingers are restricted, in the hand of the higher classes of Vertebrata. We find, moreover, as numerous and striking modifications of the pectoral fins, in adjustment to the peculiar habits of the species in Fishes, as we do of the fore-limbs in any of the higher classes. This fin may wield a formidable and special weapon of offence, as in many Siluroid fishes. But the modified hands have a more constant secondary office, that of touch, and are applied to ascertain the nature of surrounding objects, and particularly the character of the bottom of the water in which the fish may live. You may witness the tactile action of the pectoral fins when gold fish are transferred to a strange vessel: their eyes are so placed as to prevent their seeing what is below them; so they compress their air-bladder, and allow themselves to sink near the bottom, which they sweep as it were, by rapid and delicate vibrations of the pectoral fins, apparently ascertaining that no sharp stone or stick projects upwards, which might injure them in their rapid movements round their prison. If the pectorals are to perform a special office of exploration certain digits are liberated from the web, and are specially endowed with nervous power for a finer sense of touch, as we see in the gurnards, where they compensate for the loss of the tactile property consequent on the hard covering of the exterior of the mouth in these mailed-cheeked fishes." P. 155.

